# Evaluation of a Knowledge Mobilization Campaign to Promote Support for Working Caregivers in Canada: Quantitative Evaluation

**DOI:** 10.2196/44226

**Published:** 2023-06-22

**Authors:** Sarah E Neil-Sztramko, Maureen Dobbins, Allison Williams

**Affiliations:** 1 National Collaborating Centre for Methods and Tools McMaster University Hamilton, ON Canada; 2 Department of Health Research Methods, Evidence & Impact McMaster University Hamilton, ON Canada; 3 School of Nursing McMaster University Hamilton, ON Canada; 4 School of Earth, Environment & Society McMaster University Hamilton, ON Canada

**Keywords:** informal caregiver, knowledge mobilization, social media, workplace standard

## Abstract

**Background:**

As population demographics continue to shift, many employees will also be tasked with providing informal care to a friend or family member. The balance between working and caregiving can greatly strain carer-employees. Caregiver-friendly work environments can help reduce this burden. However, there is little awareness of the benefits of these workplace practices, and they have not been widely adopted in Canada. An awareness-generating campaign with the core message “supporting caregivers at work makes good business sense” was created leading up to Canada’s National Caregivers Day on April 5, 2022.

**Objective:**

Our primary objective is to describe the campaign's reach and engagement, including social media, email, and website activity, and our secondary objective is to compare engagement metrics across social media platforms.

**Methods:**

An awareness-generating campaign was launched on September 22, 2021, with goals to (1) build awareness about the need for caregiver-friendly workplaces and (2) direct employees and employers to relevant resources on a campaign website. Content was primarily delivered through 4 social media platforms (Twitter, LinkedIn, Facebook, and Instagram), and supplemented by direct emails through a campaign partner, and through webinars. Total reach, defined as the number of impressions, and quality of engagement, defined per social media platform as the engagement rate per post, average site duration, and page depth, were captured and compared through site-specific analytics on Facebook, Instagram, Twitter, and LinkedIn throughout the awareness-generating campaign. The number of views, downloads, bounce rate, and time on the page for the website were counted using Google Analytics. Open and click-through rates were measured using email analytics, and webinar registrants and attendees were also tracked.

**Results:**

Data were collected from September 22, 2021, to April 12, 2022. During this time, 30 key messages were developed and disseminated through 74 social media tiles. While Facebook posts generated the most extensive reach (137,098 impressions), the quality of the engagement was low (0.561 engagement per post). Twitter resulted in the highest percentage of impressions that resulted in engagement (24%), and those who viewed resources through Twitter spent a substantial amount of time on the page (3 minute 5 second). Website users who visited the website through Instagram spent the most time on the website (5 minute 44 second) and had the greatest page depth (2.20 pages), and the overall reach was low (3783). Recipients’ engagement with email content met industry standards. Webinar participation ranged from 57 to 78 attendees.

**Conclusions:**

This knowledge mobilization campaign reached a large audience and generated engagement in content. Twitter is most helpful for this type of knowledge mobilization. Further work is needed to evaluate the characteristics of individuals engaging in this content and to work more closely with employers and employees to move from engagement and awareness to adopt caregiver-friendly workplace practices.

## Introduction

In 2018, a total of 7.8 million (roughly 1 in 4) Canadians over the age of 15 were responsible for providing informal care to a family or friend with a long-term health condition, physical or mental disability, or age-related issues [1]. These caregivers provide an estimated 5.7 billion hours of unpaid care per year, equivalent to 2.8 million full-time jobs and an estimated CAD $97.1 billion (US $72.2 billion; a currency exchange rate of CAD $1=US $0.74034 is applicable) [2]. The role of informal caregiving is perceived to be a short-term experience, for example, time-limited care for an aging parent at the end of life; however, compressed generational care (mean duration of 3.8 years) represents the experience of only 54% of carers [3]. A population-based analysis found that for 25% of carers, the care trajectory is broad, lasting an average of 13.8 years; for 11% of carers that report intensive parent care, the average duration was 10.9 years [3]. By 2031, the proportion of older adults who are older than 65 years is projected to double due to the aging population [4]. As this population segment grows, there will be an accompanying increase in health care resources and an increasing need for informal care from family or friends. Consequently, the aging population poses challenges for the health care system and the Canadian economy and workforce, given the high proportion (over 35%) of the workforce who have caregiving obligations; these workers are defined as carer-employees [5].

Balancing paid work and caregiving can be a tremendous task. While research supports life satisfaction and psychosocial benefits from providing care, this is predominantly restricted to those not formally employed [6]. Research has shown that carer-employees are at higher risk of several adverse health outcomes due to caregiver burden and the struggle to maintain a healthy work-life balance; these include a range of physical and mental health outcomes, such as stress, depression, sleep loss, and muscle pain balance [6-8]. Lack of workplace support also impacts employers regarding reduced productivity, turnover, and absenteeism. In a nationwide survey of carer-employees, 25% had taken a sick day and 50% had taken a vacation day to attend to caregiving needs; note that 29% reported reducing their work hours, 17% have changed from full to part-time, and 15% have taken a leave or left their job altogether due to caregiving responsibilities [9].

Given the significant impact on employees, employers, and the health care system, caregiver-friendly workplace practices are needed to accommodate carer-employees while sustaining workplace efficiencies. These practices have been shown to help enhance work-life balance and workforce retention and reduce health care costs [10]. The most common caregiver-friendly workplace practices include flexible work schedules, the ability to use vacation or paid sick leave to provide care, and job-protective leave (at periods beyond the government-mandated time frame) [11,12]. Despite their potential benefits, many companies do not currently offer caregiver-friendly workplace practices due to the lack of initiative on the part of employers, the perceived challenges of implementing, or organizational limitations, such as a small staff or a lack of human resources personnel. Of the 5000 Canadian employees surveyed, only 12% reported that their organization had formal caregiving policies in place [13,14]. Lack of workplace support through family-friendly workplace practices can result in carer-employees leaving the workforce or missed workdays, early retirements, reduced productivity, health consequences, and increased avoidable costs to employers [14].

To address this issue, a team at McMaster University partnered with the Canadian Standards Association (CSA), among others, to create a bilingual CSA B701-17 Caregiver Inclusive and Accommodating Workplace Organizations Standard for Canadian workplaces (referred to herein as the Standard) [15,16], together with a handbook, B701HB-18: Helping worker-carers in your organization [17], through a Healthy Productive Work Partnership Development grant. Alongside a suite of other resources targeting carer-employees and employers [18], the Standard also included an easy-to-use, practical tool to assist employers in managing the issues that inevitably arise for individual employees as they face the growing demands associated with becoming an informal caregiver [19]. The Standard aims not only to support a work-life balance policy but to position the carer policy as a strategic business decision aimed at improving company loyalty and aiding in recruiting and retaining talented employees [11].

A primary focus of this initiative was to mobilize knowledge and disseminate the Standard nationally and internationally. As part of a multicomponent knowledge mobilization strategy, a digital campaign leading up to National Caregivers Day was created to increase awareness of carer-employees' needs and share the suite of tools developed as part of the Healthy Productive Work Partnership Development grant. Due to the growing use of social media by the public, researchers and practitioners are increasingly turning to web-based knowledge mobilization methods to increase the dissemination of their work beyond traditional academic conferences and publication [20,21]. To generate awareness about carer-employees’ needs, the availability of the newly published Standard, and its accompanying tools, we launched a knowledge mobilization campaign in partnership with Carers Canada, leading up to National Caregivers Day in Canada. This study aims to describe the reach and impact of the Supporting Working Caregivers campaign and compare the reach and engagement associated with 4 social media platforms.

## Methods

### The Campaign

**Figure 1:**
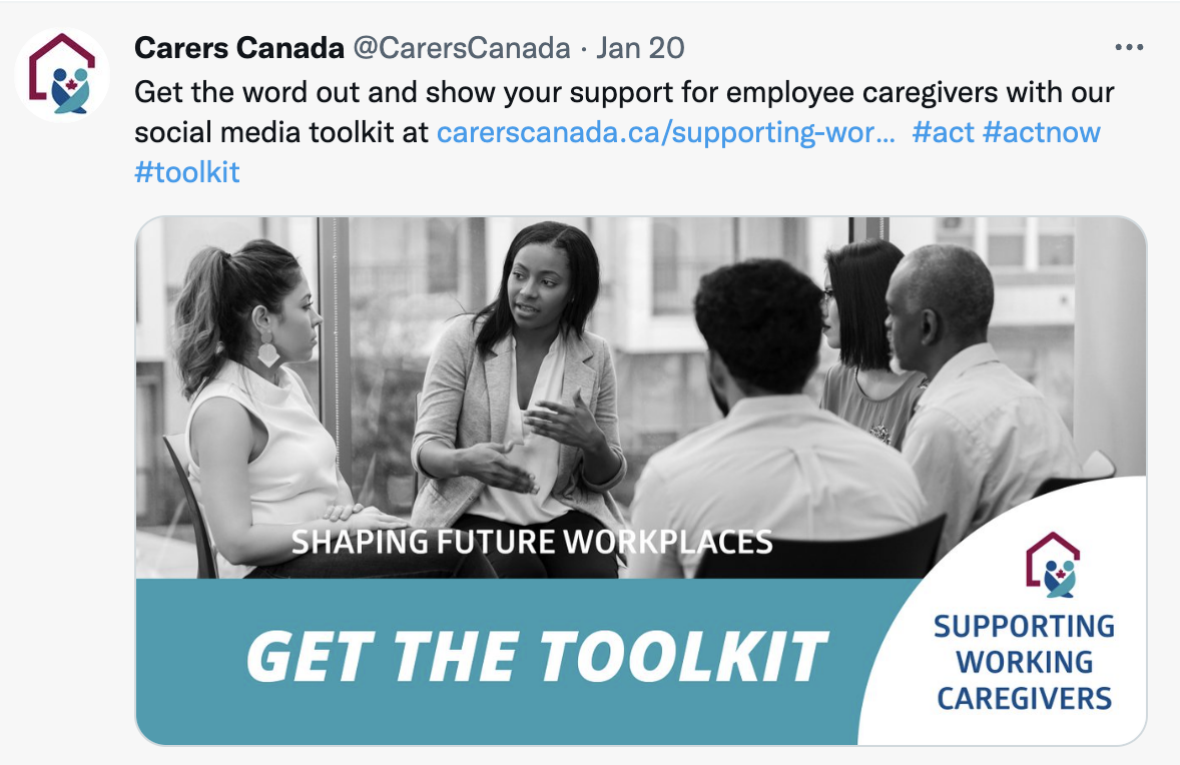
Sample social media tile.

The goal of the knowledge mobilization campaign was to build awareness of the need for caregiver-friendly workplaces and to direct workers and employers to relevant resources. A broad audience was targeted by engaging partners, creating messages, and sharing content through 4 social media platforms, Twitter, LinkedIn, Facebook, and Instagram. Social media content was delivered in partnership with a paid marketing agency with the goal of directing individuals to the campaign site. All content was disseminated organically, with the exception of 2 paid posts on each platform. This was supplemented by direct email communications from an organizational partner, Carers Canada. The core message, “supporting caregivers at work makes good business sense,” was reinforced through messages that promoted the facts, concepts, research, and solutions relevant to carer-employees and caregiver-friendly workplaces. All social media and email communications were linked to a campaign website, which included facts, statistics, employer resources, and a communications toolkit to support individual and organization advocacy. To resonate with the target audiences of both carer-employees and employers, the knowledge mobilization campaign profiled the experiences of carer-employees and employee champions who were partners with the funded partnership grant (Figure 1). Evidence blogs (n=18) were created to promote findings from the various projects within the Healthy Productive Work Partnership Development grant. A series of webinars (n=3) were hosted to share the campaigns key messages and generate awareness of the resources and tools available to employees and employers. The campaign focused on building momentum toward Canada’s National Caregivers Day on April 5, 2022.

### Data Collection and Analysis

The first goal to increase awareness was engagement through social media and email blasts. The number of posts across social media platforms and the price paid per promoted post were tracked throughout the campaign. Engagement outcomes were extracted for each platform, including total impressions (defined by each social media platform), social media profile visits, and click rates on content. The open and click rates were captured for each email using email analytics and the number of new newsletter subscribers. Webinar registrants and attendees were counted. A second step to awareness generation is converting a social media engagement to a visit to the campaign website. Unique users, average page depth, and average time on the campaign website were tracked using Google Analytics. The number of links clicked on the website and resource downloads were also tracked.

### Ethical Considerations

As no individual participants were enrolled in the study and no individual data were collected, human subjects ethics review was not required in this study.

## Results

The Supporting Working Caregivers: Shaping Future Workplaces campaign was launched on September 21, 2021, culminating in National Caregivers Day on April 5, 2022. All data were captured on April 11, 2022. A total of 30 key messages were developed and disseminated through 74 social media tiles with text on all 4 platforms and 3 videos from key Canadian government officials.

On social media, the campaign resulted in 231,062 impressions, 21,562 unique views of content, and 2510 visitors to the campaign site. Impressions varied widely across platforms, ranging from 137,098 through Facebook to 3783 through Instagram (Table 1). While Facebook reached the largest number of users, the conversion to profile visits was highest on Twitter (4.1 impressions for 1 profile visit) and lowest on Facebook (178.3 reaches for 1 profile visit). This variation was also seen across the paid advertisements, with the average cost per click across all campaign posts highest on LinkedIn (CAD $6.10 [US $4.54]) and lowest on Twitter (CAD $0.19 [US $0.14]).

**Table 1:** Breakdown of reach and engagement through 4 social media platforms, from September 21, 2021, to April 11, 2022.

		Facebook		Twitter		LinkedIn		Instagram
**All posts**								
	Impressions	137,098	81,800		8381		3783^a^	
	Profile visits	769	20,101		374		318^a^	
	Ratio of impressions to profile visits	178.3	4.1		22.4		11.9^a^	
	Engagement rate per post (%)	0.561	24.573		4.462		8.406^a^	
	Average site duration (min:sec)	00:27	3:05		1:49		5:44^b^ and 1:08^c^	
	Page depth (pages)	1.22	1.89		1.65		2.20^b^ and 1.71^c^	
**Paid posts only (CAD $)^d^**								
	Total spend	786.44^e^	633.95		4 767.24		786.44^a,e^	
	Cost per click	0.92	0.19		6.10		0.88^a^	

^a^Stories+grid.

^b^Stories only.

^c^Grid only.

^d^CAD $1=US $0.74034.

^e^Paid advertising on Facebook and Instagram are combined.

**Figure 2:**
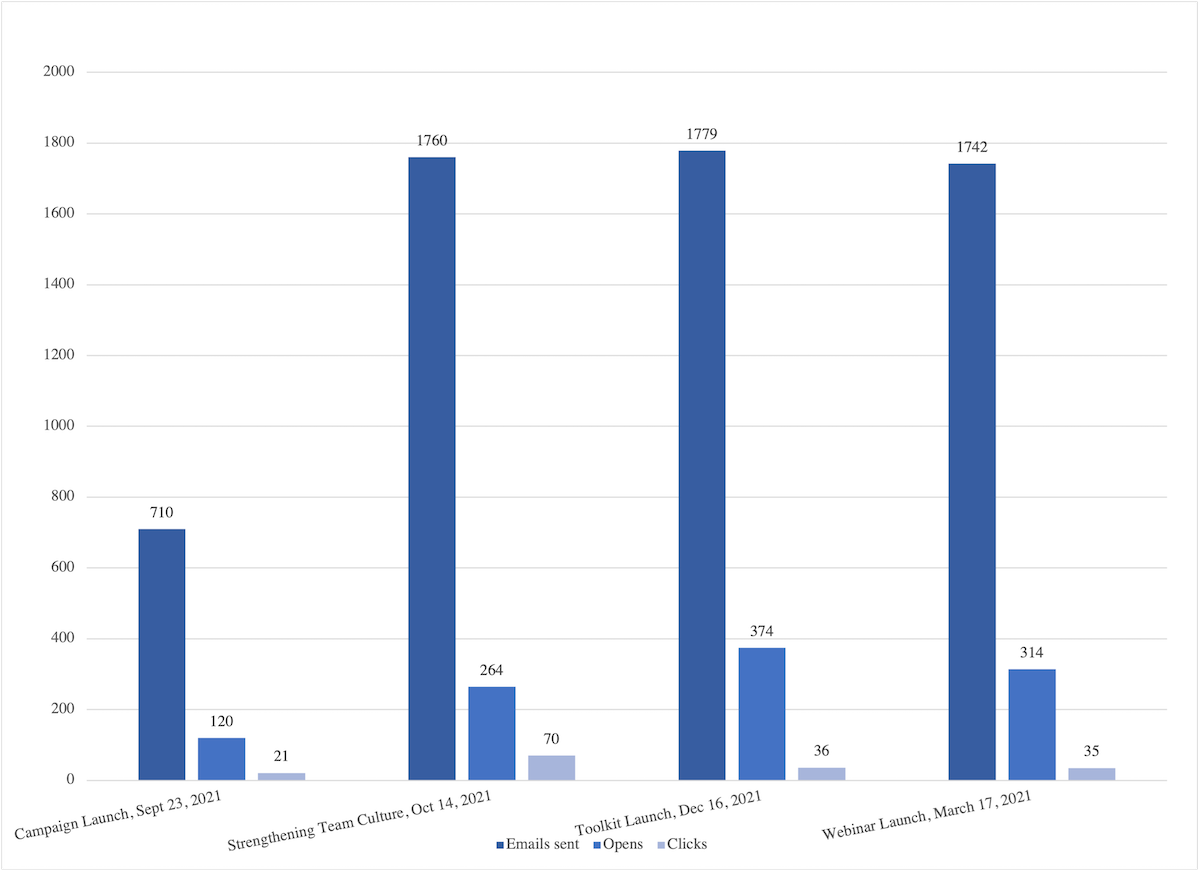
Email metrics throughout the campaign.

The email campaign consisted of 4 targeted emails from September 2021 to March 2022 highlighting the campaign launch, promoting the first webinar, highlighting the toolkit launch, and promoting the final 2 webinars held in advance of National Caregiver Day. Email subscribers, open rates, and click-through rates are displayed in Figure 2. While email subscribers increased following the campaign launch, open and click-through rates remained constant throughout the campaign, averaging 17.6% and 2.6%, respectively. A total of 3 webinars were hosted throughout the campaign, which aimed to share knowledge about the need for caregiver-friendly workplaces, and resources to support both carer-employees and employers. Webinar registration ranged from 90 to 118 (average 107) registrants and 57 to 78 (average 68) attendees.

**Figure 3:**
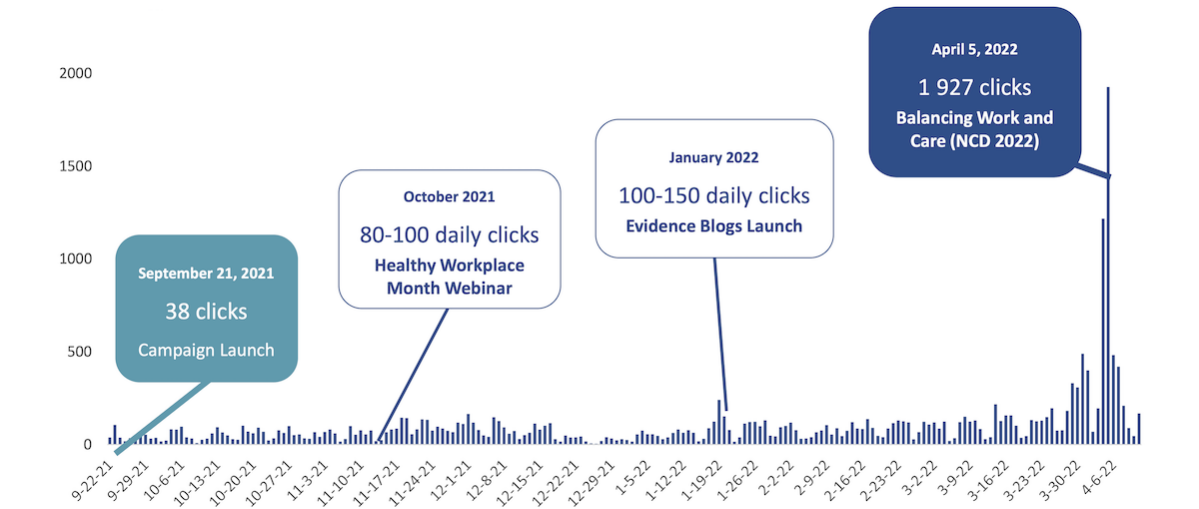
Site traffic over time: daily total clicks.

As a result of the knowledge mobilization campaign, traffic to the campaign website fluctuated throughout the campaign, increasing in the final month in the lead-up to National Caregiver Day (Figure 3). Apparent increases were seen in response to webinars (eg, October 2021) and the launch of the evidence blogs in January 2022. Across 2510 unique visitors to the website, there were 2424 trackable events, which consisted of 1510 external link clicks and 875 downloads. The top downloads were the National Caregiver Day Key Messages (65 downloads), Quick Start Implementation Guide (46 downloads), and Supporting Working Caregivers Infographic (44 downloads). The average time spent on the website was 2 minutes 4 seconds. The campaign effectively spread awareness of the tools to a new audience; 88% of visitors to the organization's website during the campaign were new users and 12% were returning users. Users were primarily from Canada (80%), followed by the United States (11%), and were predominantly English speakers (92.5%). Access from a desktop was most common (62.7%), followed by mobile (34.4%) and tablet (2.9%); bounce rates (ie, the percentage of visitors that leave the site after viewing only 1 page) were the highest for mobile users. Throughout the campaign, 34% of users came through a search, 33% direct, 17% through social media, and 16% through various other referral sources.

## Discussion

In this study, we describe the reach and engagement that resulted from a targeted knowledge mobilization campaign aiming to generate awareness of the importance of a caregiver-friendly workplace and tools and resources to support carer employees and employers. The campaign resulted in 231,062 social media impressions, 21,562 unique views of content and 2510 visitors to the campaign site. Overall, the campaign successfully drove clicks to the site, with an apparent increase in usage around critical milestones, such as webinars and launching new products, such as blog posts. User engagement with the site was extremely high, and 97% of visitors either visited a subsequent page or downloaded a resource.

A total of 72% of social media clicks came from Facebook, and this was primarily the result of the high reach due to boosted content. Indicators of the quality of engagement, such as time per page of users who visited the site through a given social media platform, were highest through Instagram at an average of 5 minutes 44 seconds on the site; however, due to the small reach, the overall impact of Instagram was low, suggesting that this platform may not be the strongest for knowledge mobilization for this target audience. Given that the topic was most relevant to the workplace, we anticipated that LinkedIn would generate the highest quality engagement. However, the ratio of impressions to profile visits, time spent on website, and page depth were inferior to Twitter. When combined with the cost per click data of Twitter (CAD $0.19 [US $0.14]) compared to LinkedIn (CAD $6.10 [US $4.54]), Twitter may be the preferred platform for future dissemination. Interestingly, the engagement rates found in this study were much higher than industry standards on Facebook (0.561% vs 0.114%), Twitter (24.573% vs 0.054%), and Instagram (8.406% vs 1.039%), respectively [22]. A number of factors can influence variation in impressions, including the size of the audience for the given platform, the budget for paid advertising, the advertisements objectives, the differences in the advertisements, etc. As a partner marketing firm was responsible for delivering the knowledge mobilization campaign, some of these details are unavailable. It is likely that the most appropriate platform would vary based on the campaign’s target audience. While these findings may not be generalizable to other knowledge mobilization campaigns, we believe they add to the body of knowledge about the use of social media for researchers and others looking to disseminate their findings beyond the academic community.

While the number of email subscribers increased during the campaign, the open and click rates remained constant. However, engagement with email was average, as both numbers align with benchmarks in this area (17.6% vs 21.5% for open rate, and 2.6% vs 2.3% for click rate) [23]. Webinar engagement exceeded industry benchmarks: 65% of webinar registrants attended versus the 50% industry standard [24].

The findings from this campaign are comparable to others that have been reported in the literature. A team in Australia led a 4-month digital and social marketing campaign to drive traffic to a website to address comorbid physical and mental health disorders; they reported an average time on page of 2 minutes 28 seconds [25]. Like our campaign, Twitter was the most effective form of engagement, and users were predominantly accessing content through desktop (72.1%) rather than phones or tablets (27.9%) [25]. A similar social media campaign to increase awareness of pain in dementia, using Twitter alone, generated over 5 million impressions over a 5-month period, which resulted in 1218 unique website visitors [26]. One of the most successful social media campaigns for knowledge mobilization comes from Chambers’ #ItDoesntHavetoHurt initiative. Over 12 months, the campaign resulted in over 130 million content views, and the campaign hashtag is still active today [27]. While variations in target audience and type of content make it unreasonable to make head-to-head comparisons across campaigns in terms of total reach and number of engagements, the high proportion of clicks per engagement and number of unique website visitors support campaign goals that were achieved.

While researchers are increasingly turning to social media to share research findings, it is unknown whether these methods of knowledge mobilization are effective. It is widely accepted that engaging with digital content is not a direct indicator of adoption or implementation. We acknowledge that viewing the campaign website is many steps removed from an employer embracing carer-friendly workplace policies; this is a limitation in this study. However, the goal of this campaign was to generate awareness about the burden that carer-employees face and the positive impact generated not only on employees but also employers and the overall workforce by embracing carer-friendly workplace policies. Indeed, the next step in this work is to engage more closely with employers to promote the adoption and implementation of the Standard created as part of the Healthy Productive Work Partnership.

### Conclusions

In conclusion, this study describes the reach and engagement of a knowledge mobilization campaign, including social media, direct email, and webinars, to increase awareness of the importance of carer-friendly workplace policies. Through this campaign, leading up to National Caregiver Day, our team saw substantial engagement with social media content, high conversion to interaction with the campaign website, and numerous downloads of knowledge mobilization tools and resources on the campaign website. We hope that this awareness-generating campaign is an important first step. However, more work must be done to ensure that employers and organizations move from information awareness to action and consider adopting workplace standards to support carer employees in Canada and beyond.

## Abbreviations

CSA: Canadian Standards Association

## Data Availability

Data are available upon reasonable request.
